# Pediatric infectious disease physician perceptions of antimicrobial stewardship programs

**DOI:** 10.1017/ice.2022.183

**Published:** 2023-07

**Authors:** Jason G. Lake, Michael J. Durkin, Philip M. Polgreen, Susan E. Beekmann, Adam L. Hersh, Jason G. Newland

**Affiliations:** 1 Division of Pediatric Infectious Diseases, Department of Pediatrics, University of Utah School of Medicine, Salt Lake City, Utah; 2 Division of Adult Infectious Diseases, Department of Internal Medicine, Washington University in St Louis, St Louis, Missouri; 3 Department of Epidemiology, College of Public Health, University of Iowa, Iowa City, Iowa; 4 Department of Internal Medicine, University of Iowa, Iowa City, Iowa; 5 Division of Infectious Diseases, Department of Pediatrics, Washington University in St. Louis, St. Louis, Missouri

## Abstract

Pediatric antimicrobial stewardship programs (ASPs) improve antibiotic use for hospitalized children. Prescriber surveys indicate acceptance of ASPs, but data on infectious diseases (ID) physician opinions of ASPs are lacking. We conducted a survey of pediatric ID physicians, ASP and non-ASP, and their perceptions of ASP practices and outcomes.

Antimicrobial stewardship programs (ASPs) are integral to addressing antibiotic overuse and resistance. Pediatric ASPs have been shown to decrease inappropriate antimicrobial use,^1,2^ drug costs, and prescription errors,^1,2^ and to increase guideline adherence,^1,2^ without increasing adverse events such as treatment failures and hospital readmissions.^1–5^ Surveys including pediatric prescribers suggest that ASPs are generally viewed favorably.^6–9^ Data regarding the perceptions of infectious disease (ID) physicians who manage ASPs, and those of ID physicians who are not ASP participants (non-ASP), are limited to a 2008 survey of pediatric ID physicians by the Infectious Diseases Society of America Emerging Infections Network (EIN), though only 33% of respondents reported having an ASP at that time.^10^ As ASPs have become more prevalent, understanding the perceived differences in ASP and non-ASP physicians is vital because disagreement between ASP and non-ASP physicians could compromise ASP activities. The opportunities for ASP and non-ASP interactions have increased in recent years with the growth of ASP programs; thus, we conducted an EIN survey of pediatric ID physicians, ASP and non-ASP, to understand the difference in perception of ASP procedures and outcomes in their respective facilities.

## Methods

In June 2019, a 10-question multiple-choice survey was distributed to pediatric ID physicians who participate in the IDSA EIN. The survey assessed differences in perceptions of ASP interventions and outcomes between ASP and non-ASP ID physicians and the prescribing habits of other clinician groups. A Likert scale was used for most questions. Two weekly reminder e-mails were sent. Only respondents who indicated that their facility had an ASP were allowed to complete the survey. Analyses were restricted to respondents who answered at least 1 question. Mann-Whitney *U* and χ^2^ tests were used for comparisons as appropriate. Statistical analyses were performed using SAS version 9.4 software (SAS Institute, Cary, NC).

## Results

Overall, 176 (49%) of 359 pediatric ID physicians surveyed responded. Among them, 161 (91%) worked in a hospital with an ASP and answered at least 1 question; 96 indicated participating as an ASP member. Most (80%) indicated ≥5 years of postfellowship experience. Also, 64% were employed in a university or medical school setting and practiced at a university (66%) or nonuniversity teaching (26%) hospital. All US regions and some Canadian regions were represented.

ASP physicians (94%) and non-ASP physicians (81%) agreed that the ASP at their institution had improved appropriate antibiotic prescribing in the previous 2 years. Most ASP physicians (91%) and non-ASP physicians (84%) disagreed with the statement that ASP had been too focused on reducing antimicrobial costs with resultant compromise of patient care, and a larger proportion of ASP physicians strongly disagreed. Moreover, <50% of both ASP and non-ASP physicians agreed that ASPs should steward their ID colleagues (Table 1).

**Table 1.** Antimicrobial Stewardship Program (ASP) Participants Versus Non-ASP Infectious Diseases Physician Agreement With Select Statements Regarding ASP Practices in Their Respective Facility

Provider Type	Total No.	Scale, % Total Respondents						*P* Value
		Strongly Disagree	Disagree	Neutral	Not Sure	Agree	Strongly Agree	
**ASP improved overall appropriateness of antibiotic prescribing in the last 2 years**								
Non-ASP	63	0	1.6	13	4.8	41	40	.12
ASP	96	1.0	0	5.2	0	47	47	
**Stewardship program in my hospital is too focused on reducing antimicrobial costs in a way that may interfere with providing appropriate care in the last 2 years**								
Non-ASP	63	32	52	6.4	4.8	4.8	0	.04
ASP	96	48	43	3.1	2.1	3.1	1.0	
		Scale, % Total Respondents						
		Yes			No		Not Sure	
**ASPs should steward (manage) antibiotic choices of their ID colleagues**								
Non-ASP	63	43			38		19	.87
ASP	94	45			36		19	
**Collectively, the physicians/clinicians in each of these groups at my institution prescribe antibiotics according to ASP principles (eg, right drug, right dose, right duration at right time)**.								
**Critical care**								
Non-ASP	63	11	33	21	3.2	30	1.6	.03
ASP	96	5.2	27	18	1.0	45	4.2	
**Surgery**								
Non-ASP	63	11	43	29	3.2	14	0	.36
ASP	95	9.5	40	25	2.1	23	0	
**Hospitalists**								
Non-ASP	62	1.6	8.1	19	4.8	60	6.5	.01
ASP	96	2.1	3.1	10	2.1	67	16	
**Infectious diseases**								
Non-ASP	62	0	1.6	4.8	0	44	50	.65
ASP	96	1.0	1.0	2.1	0	51	45	
**Oncology**								
Non-ASP	63	9.5	32	25	3.2	29	1.6	.10
ASP	95	7.4	26	18	3.2	42	3.2	
**Neonatology**								
Non-ASP	61	3.3	16	30	3.3	41	6.6	.64
ASP	95	6.3	18	21	1.1	41	13	

ASP physicians were more likely than non-ASP ID physicians to agree that critical care (49% vs 32%; *P* = .03) and hospitalists (83% vs 67%; *P* = .01) adhered to ASP principles. More than 50% of both groups disagreed with the statement that surgeons adhered to ASP principles (Table 1).

Most physicians in both groups were not concerned that ASPs make recommendations without seeing patients. Furthermore, ∼30% of non-ASP and 23% of ASP physicians were at least somewhat concerned about disagreement between ASP and ID physicians (*P* = .77). Most ASP and non-ASP ID physicians were either neutral or were not concerned about impact on prescriber autonomy, unintended consequences of guidance, decreases in prescriber efficiency, or delays in antibiotic initiation (Table 2).

**Table 2.** Antimicrobial Stewardship Program (ASP) Participants Versus Non-ASP Infectious Diseases Physician Concern Regarding ASP Practices and Outcomes in their Respective Facility

Provider Type	Total No.	Scale, % Total Respondents				*P* Value
		Not Concerned	Neutral	Somewhat Concerned	Very Concerned	
**ASP recommends without seeing patients**						
Non-ASP	62	71	16	13	0.0	.39
ASP	96	66	16	16	3.1	
**Disagreement between ASP and ID**						
Non-ASP	63	54	16	24	6.3	.77
ASP	95	53	24	19	4.2	
**ASP decreases prescriber autonomy**						
Non-ASP	63	62	22	13	3.2	.33
ASP	96	56	18	24	2.1	
**Unintended consequences of ASP guidance**						
Non-ASP	63	76	14	9.5	0.0	.70
ASP	96	73	19	7.3	1.0	
**ASP decreases prescriber efficiency**						
Non-ASP	63	76	14	9.5	0.0	.23
ASP	96	73	19	7.3	1.0	
**ASP delays antibiotic initiation**						
Non-ASP	63	62	25	11	1.6	.37
ASP	95	69	19	11	1.1	

## Discussion

Overall, both ASP and non-ASP physicians viewed ASPs favorably, and concerns about ASP practices and patient outcomes were minimal. Consistent with results from prescriber surveys,^7–9^ physicians in both groups agreed that ASPs had improved appropriate antibiotic prescribing in the prior 2 years. Reduction in unnecessary antibiotic spending should be an ASP goal,^1^ and neither group demonstrated concern that their ASP was too focused on cost reduction at the expense of patient care.

ASP physicians were more likely than non-ASP physicians to report that ID and other clinician groups prescribed antibiotics according to ASP principles. The discrepancy may arise as non-ASP physicians observe prescribing practices most often in the context of consultation, whereas ASP physicians regularly review prescribing patterns of other pediatric subspecialties and provide a less biased sample. Furthermore, ASP physicians are more likely to interact with prescribers from other specialties, resulting in an overall more favorable impression of these groups.

Almost 60% of each group disagreed or were unsure whether ASPs should steward their ID colleagues. Furthermore, non-ASP physicians were most concerned with disagreement with the ASP. In a recent survey,^9^ prescribers expressed similar concerns regarding disagreement between ASP and ID consultants. Although this could pose a significant barrier to effective ASP implementation, data are lacking on how frequently ASPs advise their ID colleagues or give conflicting recommendations. Greater collaboration between ASPs and ID physicians is needed.

Consistent with other research,^2–5,7^ <10% of respondents in either group reported concern over unintended consequences of ASP recommendations. In a survey of 153 pediatric providers (93 respondents), 94% reported never having a patient with an adverse event because of an ASP intervention.^7^ In a review of 350 ASP recommendations at a freestanding children’s hospital, there were no differences in 30-day readmission rates or length of stay (LOS) when an ASP recommendation was followed.^5^ In a separate study in the same institution, ASP recommendations for medicine patients with complex chronic conditions were associated with a lower 30-day readmission rate (4.2% vs 7.3%; *P* = .005).^4^ In a third study in the same institution among patients in hematology-oncology, neonatal intensive care, and pediatric intensive care units, a 28% decrease in the odds of death (95% CI, 0.54–0.96) was observed when a recommendation was made with no increase in readmissions or hospital-acquired *Clostridioides difficile* infection rates.^3^ Although larger, multicenter studies of patient outcomes are needed, the results of this study and others show that concern about the negative consequences of ignoring ASP recommendations is limited.

Prescriber surveys suggest differing views on how ASPs impact efficiency. Only 3.2% of ASP physicians and 6.3% of non-ASP physicians were concerned about decreasing prescriber efficiency. However, a larger proportion of both, 12% and 13%, respectively, were concerned about delays in antibiotic initiation. In a recent, single-institution survey,^9^ 140 (95%) of 145 respondents indicated that their ASP facilitated appropriate antimicrobial use, and 133 (92%) of 144 indicated that their ASP improved quality of patient care. However, 38 (26%) of 146 respondents indicated that the ASP reduced their efficiency and some expressed concerns over delays in antibiotic administration in free-text responses. Notably, the ASP at the institution in that study required prior authorization for >50 antimicrobials. However, institutions with ASPs that primarily audit antimicrobial use prospectively and provide feedback may have a different experience.

This study had several limitations. With a response rate of <50%, results may not be representative of all North American pediatric ID physicians. A larger proportion of respondents were ASP-affiliated; thus, motivation to complete the survey likely differed between the 2 groups, possibly limiting our ability to detect differences. Lastly, these data precede the SARS-CoV-2 pandemic, so results might differ were the survey conducted now.

In this survey of ASP and non-ASP pediatric ID physicians, both groups reported improvement in appropriate antibiotic prescribing and minimal negative consequences. Some differences existed over perception of other subspecialty prescribing practices and ASP outcomes, though responses were overall similar between groups. Studies to better characterize ASP practices, evaluate outcomes, and improve ASP collaborations with ID physicians are needed.
